# The effect of ranibizumab and aflibercept treatment on the prevalence of outer retinal tubulation and its influence on retreatment in neovascular age-related macular degeneration

**DOI:** 10.1186/s12886-018-0960-6

**Published:** 2018-11-14

**Authors:** Attila Kovacs, Timea Kiss, Ferenc Rarosi, Gabor M. Somfai, Andrea Facsko, Rozsa Degi

**Affiliations:** 1 Department of Ophthalmology, Faculty of Medicine, University of Szeged, 10-11 Koranyi fasor, Szeged, 6720 Hungary; 2 Department of Medical Physics and Informatics, Faculty of Medicine, University of Szeged, Szeged, Hungary; 3 Augenzentrum Pallas Kliniken, Olten, Switzerland; 4 Department of Ophthalmology, Faculty of Medicine, Semmelweis University, Budapest, Hungary

**Keywords:** Outer retinal tubulation, Prevalence, Anti-VEGF, Aflibercept, Retreatment, Subretinal hyperreflective material

## Abstract

**Background:**

We aimed to analyze the differences in the prevalence of outer retinal tubulation (ORT) in neovascular age-related macular degeneration (AMD) treated with anti-vascular endothelial growth factor (anti-VEGF) agents, either aflibercept or ranibizumab. Our further aim was to examine the changes in the frequency of injections of ranibizumab before and after ORT appearance.

**Methods:**

Two hundred thirty six eyes of 230 patients were included in the study (184 eyes treated with ranibizumab by pro re nata regimen (PRN), 52 eyes with aflibercept bimonthly) and followed for 6–24 months. Using optical coherence tomography (OCT), the first appearance of ORT was documented, and fixed time point evaluations were also made every six months to determine the existence of ORT. The number of injections, the presence or absence of subretinal hyperreflective material (SHRM) at treatment initiation and visual acuity were also noted.

**Results:**

The survival analysis with Cox proportional hazard model showed no significant difference between the ranibizumab and aflibercept groups in relation to the development of ORT (*p* = 0.79, hazard ratio 0.92). In the PRN treated ranibizumab group the number of injections showed significant decrease after ORT development (*p* = 0.004). When SHRM was present at treatment initiation the chance of developing ORT was 2.75 and 11.14 times higher in the ranibizumab and aflibercept groups, respectively.

**Conclusions:**

The prevalence of ORT increased over time independently from the chosen anti-VEGF drug. Our results suggest that upon the appearance of ORT a decrease in retreatments can be expected.

## Background

Outer retinal tubulation (ORT) is a spectral-domain optical coherence tomography (SD-OCT) biomarker [1], first described by Zweifel et al. [2]. They defined ORTs as hyporeflective, branching tubular structures with hyperreflective borders within the outer nuclear layer of the retina [2]. The “en face” OCT technique can help map these branching networks [3]. ORTs have been observed in many retinal diseases, including exudative age-related macular degeneration (AMD) [2]. Based on histological reports, the border of the outer retinal tubulation consists of photoreceptor inner segment mitochondria and external limiting membrane (ELM), with fluid and photoreceptor outer segments being potentially present in the ovoid hyporeflective lumen of the ORT [4–6]. Adaptive optics scanning laser ophthalmoscopy findings are in correlation with histology reports and show lack of ORT cone reflectivity which can be due to the loss of cone outer segments and subsequent retinal remodeling [7].

Schaal et al. classified outer retinal tubulations as either open (incomplete closure with curving external limiting membrane at the ends, horizontally elongated shape in cross-section) or closed (completely encircled, oval shape in cross-section) ORTs [4].

ORT can be mistaken for intraretinal cysts, or subretinal fluid but with the recognition of its hyperreflective border and special occurrence in the outer nuclear layer these mistakes can be reduced, leading to a reduction in the rate of anti-VEGF overtreatment in exudative AMD [2].

The ORT prevalence in exudative AMD is low at the time of first diagnosis but over time during anti-VEGF therapy its prevalence increases [8, 9]. The importance of ORT as an OCT biomarker for photoreceptor degeneration is due to its connection with reduced visual acuity [1, 8–10].

It has been also reported that ORTs develop above areas of subretinal hyperreflective material (SHRM) or atrophy [8, 9]. SHRM is a medium- to hyperreflective mass between the neurosensory retinal layers and retinal pigment epithelium on OCT [11]. It usually represents either a type II choroidal neovascular complex or is the consequence of an active choroidal neovascularisation, including subretinal haemorrhage and lipid or fluid exudation [1, 11, 12].

The aim of the present study was to investigate the prevalence of ORTs in eyes with neovascular AMD undergoing treatment either with ranibizumab or aflibercept. Our further aim was to examine the changes in the frequency of injections before and after ORT appearance. We also assessed the presence of subretinal hyperreflective material and its relationship with ORT.

## Methods

### Ethics, consent

This retrospective study was performed at the Medical Retina Unit of the Ophthalmology Department of University of Szeged, in Hungary. The study was approved by the Institutional Review Board of University of Szeged Albert Szent-Györgyi Clinical Centre (reference number: 3650) and was in accordance with the ethical standards of the Declaration of Helsinki. Since this was a retrospective review of patient data, informed consent was not required. The need for a consent was formally waived by the ethics committee, and this was also in line with the national regulations.

### Patients

Treatment-naïve exudative AMD patients were enrolled in the study. For the ranibizumab group enrollment took place between October 2014 to April 2016 while patients in the aflibercept group were enrolled between April 2015 to April 2016.

All patients were over 50 years of age, the mean follow-up period was 16.3 months and 9.2 months (range 6–24 months and 6–12 months) in the ranibizumab and aflibercept groups, respectively.

During each visit a comprehensive ophthalmic examination was carried out including best-corrected visual acuity (BCVA, Early Treatment Diabetic Retinopathy Study (ETDRS) score) assessment, slit-lamp biomicroscopy, dilated funduscopy and SD-OCT examination of the retina (Heidelberg Spectralis, Heidelberg Engineering, Heidelberg, Germany). Eyes with poor quality SD-OCT scans (Q index below 20) or with poor compliance were excluded from the study (14 eyes from the ranibizumab and 3 eyes from the aflibercept group).

Treatment regimen for both ranibizumab (0.5 mg) and aflibercept (2 mg) started with 3 monthly injections. After this initiation phase the ranibizumab group was treated by a pro re nata (PRN) regimen with follow-up visits scheduled monthly. The retreatment criteria for ranibizumab patients consisted of any subretinal or intraretinal fluid on OCT, or new haemorrhage on funduscopy. In the aflibercept group follow-up after the loading phase was scheduled every two months, treatment was given at each follow-up. The above regimens were in accordance with the available treatment guidelines in Hungary at the time of the study.

**Fig. 1:**
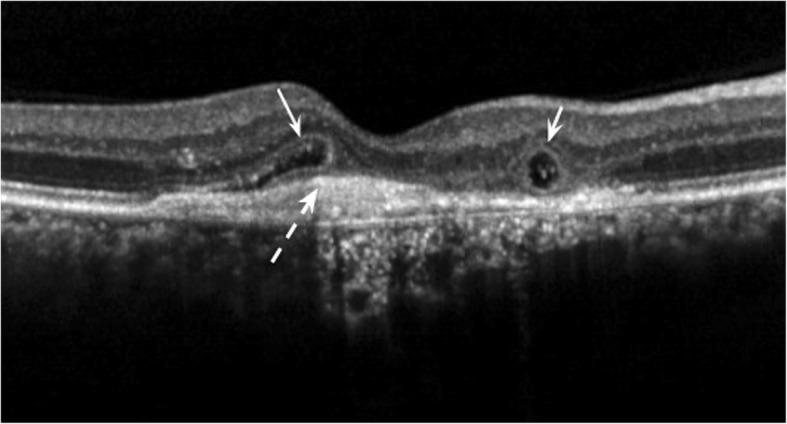
Outer retinal tubulations and subretinal hyperreflective material on an SD-OCT single B-scan. Open and closed ORTs in cross section (left and right solid arrows) above subretinal hyperreflective material (dash arrow). The definition of ORT was a hyperreflective ovoid-elongated structure in the outer nuclear layer of the retina with lower reflective content

For SD-OCT imaging a pattern size of 5.8 × 5.8 mm, 20° × 20° was applied with 25 B-scans, using the “follow-up” mode. By manual review of the scan volumes we determined the first appearance of the ORT in both groups. We also assessed the presence of ORT at fixed time points at baseline, month 6 and 12 in both groups and at months 18 and 24 in the ranibizumab group. Images were assessed by two independent retina specialists, in case of incongruity the images were referred to a third retina specialist to make a decision. During the evaluation of OCT scans we did not differentiate between the above described open (incomplete hyperreflective ring) and closed (complete hyperreflective ring) forms of ORT according to Schaal [4]. Thus, both types of ORT detected on the images were considered an ORT positive case. The criterion of ORT was a hyperreflective ovoid-elongated structure in the outer nuclear layer of the retina with lower reflective content (Fig. 1).

The readers also identified the presence or absence of subretinal hyperreflective material on SD-OCT images at treatment initiation. The criterion for SHRM was a medium- to hyperreflective mass between the neurosensory retinal layers and retinal pigment epithelium, as described by Keane et al. [11] (Fig. 1).

### Statistical methods

The BCVA was compared across the two groups using the Mann-Whitney U-test. The survival analysis for ORT development was analyzed by a Cox proportional hazard model. We analyzed the correlation between the presence of SHRM at treatment initiation with the development of ORT by Chi-square test and calculated relative risks. Where zeros were involved for the computation of relative risk, 0.5 was added to all cells, according to the previous suggestions [13].

The injection rate was calculated only in the PRN treated ranibizumab group due to the fixed 2 month therapeutic regimen of aflibercept. We assessed the injection rate only before and after the appearance of outer retinal tubulation and compared using the the Mann-Whitney U-test. In order to correct bias due to the unequal follow-up time (some patients had a higher number of injections due to the longer follow-up), we divided the follow-up time with the number of injections and calculated with monthly injections.

A *p*-value of *p* < 0.05 was taken as statistically significant. For the analyses the IBM SPSS Software (Version 22) was used.

## Results

In the ranibizumab group we evaluated 184 eyes of 179 patients, with a median age of 74 years (range 51 to 88), while in the aflibercept group there were 52 eyes of 51 patients with a median age of 75 years (range 58 to 87).

The mean baseline best corrected visual acuities in the two groups were (mean ± SD) 59.16 ± 13.9 (median 61) and 53.96 ± 13.54 (median 55.5) ETDRS letters in the ranibizumab and aflibercept group, respectively. There was no significant difference between the two groups (Mann-Whitney U-test *p* = 0.083). The BCVA at the end of the follow-up was 57.19 ± 20.19 (median 63) and 59.46 ± 15.54 (median 64) ETDRS letters in the ranibizumab and aflibercept group, respectively. There was no significant difference between the two groups (Mann-Whitney U-test *p* = 0.69).

Table 1 shows the number of eyes during the follow-up in the two groups. The number of eyes was reduced over time due to gradual enrollment in the study, thereby not every patient reached the same follow-up time. In the ranibizumab group outer retinal tubulation was observed in 17.4% of cases at baseline, in 33.7% of cases at month 6, in 45.3% of cases at month 12, and in 55.3% and in 60.8% of cases at months 18 and 24, respectively. The ORT prevalence in the aflibercept group was 23.1% at baseline, 40.4% at month 6, and 50% at month 12.

**Table 1:** Number of eyes reaching the follow-up in the ranibizumab and aflibercept treated groups

	Ranibizumab	Aflibercept
Time point	*n* (eyes)	*n* (eyes)
Baseline	184	52
at 6 months	184	52
at 12 months	161	28
at 18 months	103	0
at 24 months	51	0

Table legend: The column with “n” corresponds to the number of eyes reaching the follow-up

**Fig. 2:**
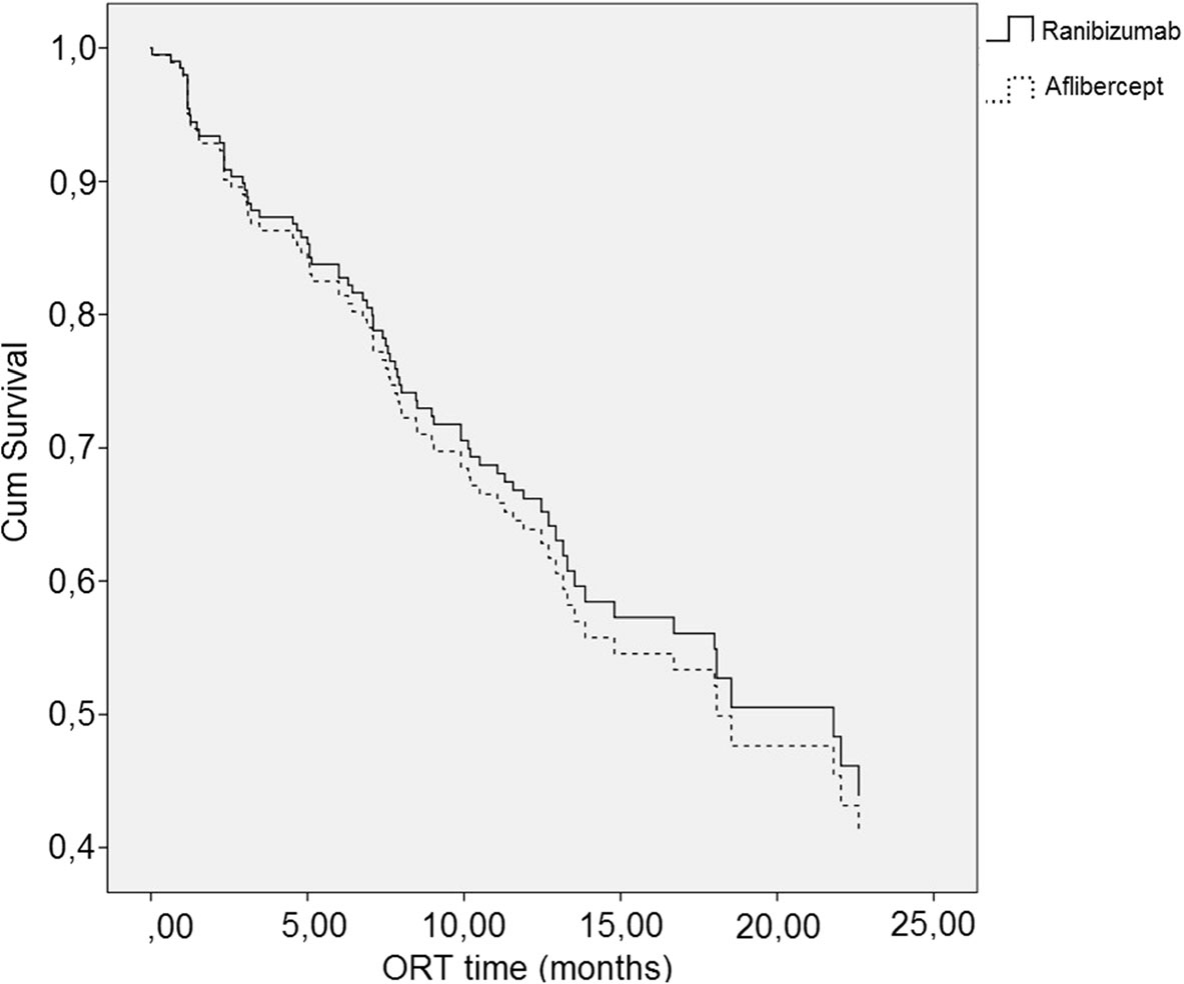
Cox proportional hazard model of ORT survival in the ranibizumab and aflibercept groups

The survival analysis showed no significant difference between the ranibizumab and aflibercept treated groups in terms of ORT development. (*p* = 0.79, hazard ratio 0.92, 95% confidence interval 0.500–1.693) (Fig. 2).

The injection rates showed that the mean injection number per month before the ORT appearance was 0.37 ± 0.17 while after the ORT development it decreased to 0.21 ± 0.17 (Mann-Whitney U-test *p* = 0.004).

The presence of subretinal hyperreflective material at treatment initiation in the two subgroups was 75.5% in the ranibizumab, and 80.8% in the aflibercept group. In the ranibizumab treated group ORT developed in 85 eyes of 139 eyes with SHRM (61.15%), while without SHRM (45 eyes) ORTs were found merely in 10 eyes (22.2%) corresponding to a relative risk of 2.75. (*p* < 0.01). In the aflibercept treated group 55.81% of eyes with SHRM developed ORT (24 eyes of 43). No ORT developed in the eyes without SHRM (out of 9 eyes), consistent with a relative risk of 11.14 (*p* < 0.01).

## Discussion

Our study found no significant difference between the ranibizumab and aflibercept treated groups according to ORT development. There was a statistically significant reduction in the frequency of injections in the ranibizumab treated group before and after ORT appearance. The presence of SHRM at treatment initiation as a biomarker had a statistically significant correlation with the development of ORT in both groups.

Zweifel first described outer retinal tubulation in 2009 [2]. Later the authors reported ORTs in various retinal disorders like neovascular AMD, geographic atrophy, polypoidal choroidal vasculopathy, non-AMD associated choroidal neovascularisation, and other degenerative retinal disorders (e.g. retinitis pigmentosa, gyrate atrophy, choroideremia, Stargardt disease, pattern dystrophy) [2, 3, 14–19].

The pathogenesis of ORT formation is still not completely clear, though recent reports helped a lot exploring it. Dolz-Marco et al. called the attention on the role of Müller cells in the pathomechanism of ORT development, namely the progressive photoreceptor damage that can result in Müller cell activation which thereby starts to produce glial fibrillary acidic protein, facilitating the formation of ORT [20]. Based on histological examinations it seems that the evolution of ORT starts with ELM and ellipsoid zone disruption [4]. ELM starts to scroll inward at its free edges, representing an initial form of ORT, leading to the development of a formed open ORT. With time the large, open ORTs split, their margins beginning to scroll ending in multiple smaller closed ORTs. During the scrolling process a downward displacement of adjacent inner nuclear layer and outer plexiform layer happens separating each ORT, and causing the appearance of microcystic lesions in the inner nuclear layer. The downward displacement of these layers might be due to the involvement of Müller cells in this scrolling/dragging process as Müller cells are contributing to the constitution of ELM with the inner segments of photoreceptors [4, 15, 20, 21].

Most of the studies in the field focused on exudative AMD and its relationship with ORT. From these reports it is known that ORT is an SD-OCT biomarker, the prevalence of which increases with time and is associated with decreased visual acuity [1, 8, 9]. It has been also reported that ORTs develop adjacent to areas of subretinal hyperreflective material or atrophy [8, 9]. The differential diagnostic importance of outer retinal tubulation lies in the avoidance of overtreatment due to its similarity to intraretinal fluid [2, 8].

In the present study, we evaluated treatment-naïve exudative AMD patients treated with ranibizumab and aflibercept regarding the presence of outer retinal tubulation. Altogether 236 eyes were followed in both groups with no statistical difference between the baseline characteristics of the two groups considering age and BCVA.

The prevalence of ORT continuously increased during the follow-up period, in both groups. In the ranibizumab group its prevalence almost quadrupled at the 24-month follow-up, while there was a doubling in the aflibercept group in 12 months. It is important to note, that in the ranibizumab group the baseline prevalence was lower (17.4% versus 23.1%). Dirani et al. found a similar increasing trend in their study starting with 2.5% at presentation, reaching 41.6% at 4 years of follow-up [8]. In our study the baseline ORT prevalence was higher compared to other reports [8, 9]. The reason behind this could be the more advanced disease state at the time of presentation (due to the real life nature of our retrospective study) and a relative delay in therapy initiation due to country-specific financial difficulties. The poorer baseline BCVA in both groups also supports this idea.

There is one previous article known, describing ORT development in 24 non-treatment-naïve eyes, treated with aflibercept only after receiving at least 6 ranibizumab injection, reporting an initial 97% ORT prevalence which later decreased to 75% [22].

To our knowledge, our study is the first to report results in treatment naïve patients treated with aflibercept and its connection with ORT development. The Cox proportional hazard model analysis suggested that there was no difference between the two in-label therapies ranibizumab and aflibercept in regard to the prevalence of outer retinal tubulation. Lee et al. in the Comparison of AMD Treatment Trials (CATT) study group evaluated the prevalence of ORTs in ranibizumab and bevacizumab treated neovascular AMD patients, and found no difference between the two drugs related to the prevalence of outer retinal tubulation [9].

In the present study we found a statistically significant difference in the monthly injection rate before and after the appearance of outer retinal tubulation in the ranibizumab treated group. Our results suggest that in patients who develop ORT a decrease in the retreatment rate can be expected which may be a very important clinical marker. Although we had a PRN regimen according to the Hungarian guidelines, Lee et al. found no difference between the fixed monthly regimen versus PRN regimen in regards to ORT development in patients treated either with ranibizumab or bevacizumab [9].

We found a statistically significant connection between ORT development and the presence of subretinal hyperreflective material at treatment initiation. When subretinal hyperreflective material was present the chance of developing ORT was 2.75., and 11.14 higher in the ranibizumab and aflibercept groups, respectively, in accordance with the results of Lee et al. in ranibizumab and bevacizumab treated patients [9].

Our findings, in concordance with the above mentioned study results suggest that ORT is independent of the chosen anti-VEGF drug or the dosing regimen of intravitreal anti-VEGF treatment. The appearance of ORT suggests that the clinicians can expect a decrease in the number of injections when following a pro re nata ranibizumab regimen. Our fixed bimonthly treatment with aflibercept did not allow us to analyze the injection rate before and after ORT development in this group. Our study also supports the previously reported higher prevalence of ORT development in the presence of subretinal hyperreflective material at treatment initiation [9].

There is a number of limitations of our study. Namely, the relatively small sample size in the aflibercept group compared to the ranibizumab group, along with the bimonthly follow-up in the aflibercept group. We believe that the number of subjects involved in both groups is comparable with other studies published in the field, while the bimonthly treatment regimen with aflibercept was fixed due to the country-specific guideline regulations. The decreasing number of eyes during the follow-up could also bias the analysis by including patients with increasing disease severity. However, we believe these factors were similar to those in similar studies available in the field. The strength of this report is the comparison of present in-label therapies, ranibizumab and aflibercept in exudative AMD patients in relation to ORT besides the evaluation of the injection rate in association with outer retinal tubulation. We used real life data that makes the study more relevant in the daily clinical practice.

## Conclusions

The development of ORT could be a potential biomarker for the treatment prognosis in patients with wet AMD. Its clinical significance lies also in its similarity to activity-related intraretinal fluid. Further studies are needed to explore the nature and development of ORTs employing a comparable dosing and follow-up regimen of all three currently available anti-VEGF drugs ranibizumab, bevacizumab and aflibercept.

## Abbreviations

AMD: Age-related macular degeneration
anti-VEGF: Anti-vascular endothelial growth factor
BCVA: Best-corrected visual acuity
CATT: Comparison of AMD Treatment Trials
CNV: Choroidal neovascularisation
ELM: External limiting membrane
ETDRS: Early Treatment Diabetic Retinopathy Study
OCT: Optical coherence tomography
ORT: Outer retinal tubulation
PRN: Pro re nata
SD-OCT: Spectral-domain optical coherence tomography
SHRM: Subretinal hyperreflective material
